# Pharmacodynamics of interspecies interactions in polymicrobial infections

**DOI:** 10.1038/s41522-024-00621-6

**Published:** 2025-01-21

**Authors:** C. Herzberg, J. G. C. van Hasselt

**Affiliations:** Leiden Academic Centre for Drug Research, Leiden University, Leiden, The Netherlands

**Keywords:** Microbial ecology, Pathogens

## Abstract

The pharmacodynamic response of bacterial pathogens to antibiotics can be influenced by interactions with other bacterial species in polymicrobial infections (PMIs). Understanding the complex eco-evolutionary dynamics of PMIs and their impact on antimicrobial treatment response represents a step towards developing improved treatment strategies for PMIs. Here, we investigated how interspecies interactions in a multi-species bacterial community affect the pharmacodynamic response to antimicrobial treatment. To this end, we developed an in silico model which combined agent-based modeling with ordinary differential equations. Our analyses suggest that both interspecies interactions, modifying either drug sensitivity or bacterial growth rate, and drug-specific pharmacological properties drive the bacterial pharmacodynamic response. Furthermore, lifestyle of the bacterial population and the range of interactions can influence the impact of species interactions. In conclusion, this study provides a foundation for the design of antimicrobial treatment strategies for PMIs which leverage the effects of interspecies interactions.

## Introduction

Polymicrobial infections (PMIs) involve the presence of multiple pathogens living in close proximity at the site of infection^1^. It has been hypothesized that interactions between bacterial species, i.e., interspecies interactions, may contribute to the risk of treatment failure in PMIs^2–4^. Interspecies interactions may alter pathogen sensitivity to antimicrobial drugs^5–8^. Moreover, these interactions can impact evolution and establishment of antimicrobial-resistant mutants^9–13^. The polymicrobial nature of various bacterial infections is increasingly recognized, in particular for respiratory tract infections in individuals with cystic fibrosis^14^, infections in patients with neutropenia^15^, blood stream infections^16^, chronic wound infections^17^, otitis media infections^3,13^ and urinary tract infections^6,18^. A better understanding of the complex dynamics of polymicrobial communities and their response to antimicrobials could help to develop enhanced therapies for patients with PMIs.

The impact of interspecies interactions on the bacterial population arises as a cumulative effect of numerous local interactions among individual bacteria. This impact depends on the strength and frequency of individual interactions. Interaction strength is inherent to the interaction’s mechanism. In contrast, interaction frequency can be shaped by various factors, including the degree of mixing between the two species and the distance at which interactions can occur. In spatially structured environments, local unmixing of species can occur as the result of growth and may limit the potential for interactions between individuals from different species at a short range^19,20^. Examples of microbial populations living in such conditions are biofilm communities, very dense populations and those living in a movement-restricting environment, such as thick mucus. Conversely, planktonic bacterial populations composed of freely floating bacteria undergo continuous interspecies mixing which may prevent local unmixing and allow for more frequent interactions. Similarly, long-range interactions may be less sensitive to spatial effects, such as local unmixing, because of their potential to reach beyond a locally unmixed region. This underlines the importance of the interaction distance and the lifestyle of bacteria for their cumulative impact on the bacterial population.

Various interspecies interactions have been identified that influence sensitivity to antimicrobial drugs. These interactions encompass intricate and often poorly understood mechanisms, involving the exchange of metabolites^6,21^, signaling molecules^13^, extracellular^5,22^ and intracellular^23^ drug inactivation, exoproduct secretion^24^, environmental shifts^25^, extracellular structures^26^, virulence factors^27^ or bacterial warfare^28^. These interaction effects can be considered as a type of collective resistance, collective tolerance, or growth enhancement and impairment strategies^9^. Although interspecies interactions may differ in their mechanisms, they often lead to similar responses in the affected bacteria, narrowing the range of relevant interactions to be considered when studying bacterial drug responses. For instance, a decrease in drug concentrations surrounding the affected species could result from drug inactivation or physical barriers that impede drug distribution. Similarly, the replication rate of a bacterial species may be altered due to changes in nutrient availability within its environment or through a metabolic shift initiated by signals from other bacterial species. In such cases, although the experiences of the affected species may differ, if the outcome—such as a change in growth behavior—is the same, the impact on drug response may also be similar. This reasoning supports a generalized approach to categorizing interactions solely based on their effect on bacterial response to antimicrobials.

The pharmacological properties of antimicrobial drugs represent another key factor which may drive the impact of interspecies interactions on the bacterial response. Specifically, the interplay between the drug target and the interaction type becomes of interest. For example, antimicrobials that target replication and whose effectiveness is proportional to the replication rate of bacteria, like rifampicin, are likely to be influenced differently compared to antimicrobials whose effectiveness is not dependent on the replication rate, such as ciprofloxacin. Thus, considering drug characteristics alongside interspecies interaction types is important to develop a more comprehensive understanding of how interactions influence the response to specific drugs. This knowledge is necessary for the development of more effective therapeutic strategies that account for the complex dynamics of PMIs.

Studying PMIs using experimental approaches, both in vitro and in vivo, presents numerous challenges due to the complex nature of PMIs and the vast array of potential copathogens. While established methods and protocols for studying bacterial response to antimicrobials primarily focus on monomicrobial populations, novel techniques are being developed to study polymicrobial populations. Theoretical approaches offer the potential to gain insights into PMIs by integrating existing knowledge of eco-evolutionary dynamics and pharmacodynamics in silico^29^. These theoretical models can inform future experimental designs and research efforts aimed at understanding how interspecies interactions impact the response of pathogens to treatment. Furthermore, in silico approaches have the potential to translate knowledge of how interspecies interactions influence pharmacodynamics into tailored treatment strategies.

In this study, we employ an in silico approach to explore the influence of interspecies interactions on the response of bacteria to antimicrobial drugs. To achieve this, we developed a model of an interactive multi-species bacterial community, combining an agent-based modeling (ABM) approach with ordinary differential equation (ODE) models. Specifically, we study two types of interspecies interactions that either transiently alter the growth or drug susceptibility of bacteria. We consider antimicrobial drugs with various pharmacological characteristics such as bactericidal, bacteriostatic, concentration-dependency and replication-dependency. Our model takes into account the heterogeneity within the bacterial population arising from these interactions, as well as the spatial distribution of individual bacteria.

We simulated unidirectional interaction scenarios between two pathogens, one of which is insensitive to drug treatment. Such a scenario can occur in polymicrobial infections where one pathogen is gram-negative and another gram-positive or when one of the pathogens is susceptible and another drug-resistant. Polymicrobial infections are usually treated with narrow-spectrum antimicrobial drugs, most of which are effective against either gram-negative or gram-positive pathogens. One well studied example of such a pathogen combination is *Pseudomonas aeruginosa* and *Staphylococcus aureus* which are common in lung infections of patients with cystic fibrosis and in chronic wound infections^30,31^. Another example is *Bacteroides fragilis* in combination with *Enterococcus faecalis* or *Clostridium perfringens* which commonly co-occur in intra-abdominal infections^32^.

We systematically evaluated how interspecies interactions influence the bacterial pharmacodynamic response to antimicrobial treatment. Additionally, we investigated how spatial effects of interspecies interactions affect the pharmacodynamic response of bacterial populations, i.e., the maximal interaction distance and bacterial lifestyles with varying degrees of cell mixing, represented by the bacterial movement speed. Overall, this comprehensive study aimed to improve our understanding of how interspecies interactions shape bacterial responses to antimicrobial drugs.

## Results

We studied the response of bacteria to antimicrobial drugs in the presence and absence of different interactions towards a focal pathogen. We simulated bacterial populations consisting of two bacterial species, a dynamic focal species and a constant secondary species. Interactions between them are affecting the focal pathogen’s drug sensitivity (i.e., MIC or maximal replication rate, called the interaction target). Interactions can increase or decrease the value of the interaction target, respectively called positive and negative interactions. To determine how interspecies interactions may influence response of the focal pathogen population to antimicrobial treatment, the focal pathogen population was analyzed at the time of maximal net growth.

**Fig. 1:**
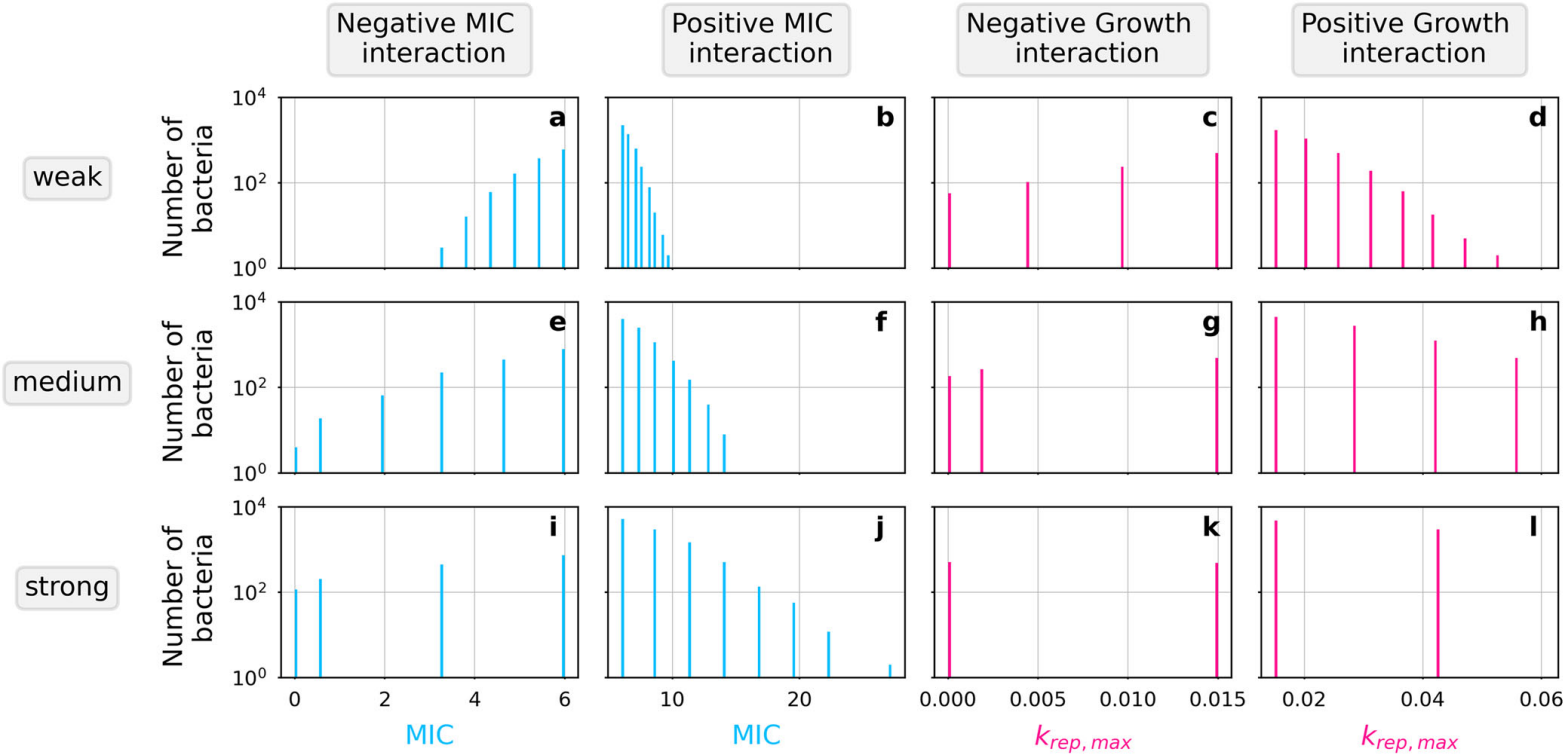
Interactions cause heterogeneity in the target population. Distributions of interaction target characteristics in the focal pathogen populations at the time of maximal net growth show the number of bacteria with range of values of the MIC and the maximal replication rate *k*_rep,max_. Populations were affected by interactions with different characteristics; interactions lowering their MIC (**a**, **e**, **i**), interactions increasing their MIC (**b**, **f**, **j**), interactions lowering their maximal replication rate (**c**, **g**, **k**), and interactions increasing their maximal replication rate (**d**, **h**, **l**). Unstructured bacterial populations were treated with a bactericidal, proportional, concentration-independent drug at a concentration of four times their MIC. Three interactions strengths of each interaction (**a–d**), weak (**e–h**), medium and strong (**i-l**), were tested individually. At the end of the timestep of maximal net growth, single cell characteristics of every bacterium which is still alive were recorded. Single-cell data from 50 iterations are presented in a combined distribution. Changes in the interaction target depend on the number of species interacting in close proximity. Each subplot shows the presence of distinct subgroups of bacteria within which all have the same value of interaction target (MIC or maximal replication rate). The subgroups can be discreetly distinguished based on how many interactions have occurred, which directly relates to the number of active bacteria within interaction distance. With increasing interaction strength, the distance between the subgroups becomes longer because each individual interaction causes a bigger change in the affected bacterium.

**Fig. 2:**
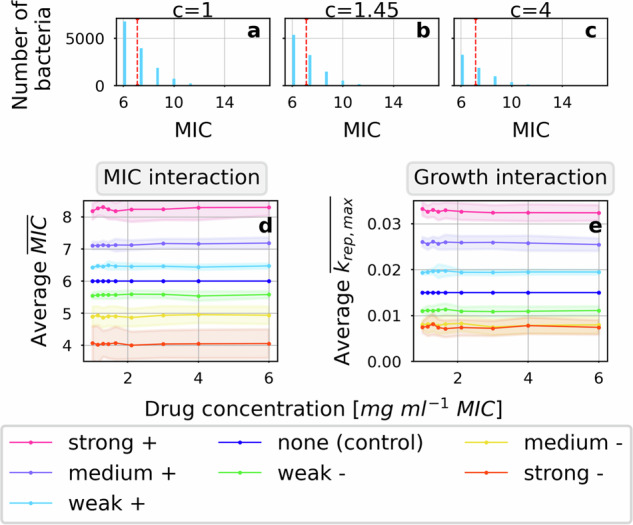
The distribution of interaction target variability in an unstructured population is similar for all above-MIC concentrations. Distributions of the interaction target characteristics in a population affected by positive MIC interactions under antimicrobial treatment at three different drug concentrations are illustrated (**a**–**c**, compare to Fig. 1f). The mean value (indicated in red) of the distributions is similar at each concentration because of the high abundance of bacteria unaffected by interactions (MIC = 6). Further, the mean of the interaction target characteristics in the focal pathogen population at the time of maximal net growth is illustrated for each drug concentration, interaction type and interaction strength (**d**, **e**). Standard deviations of the mean values between iterations are illustrated as shaded areas. The mean target was determined as the mean of 50 iterations of the mean in the population of bacteria which are still alive at the end of the timestep with maximal net growth during treatment with a bactericidal, proportional, concentration-independent drug.

The observed change in response of the bacterial populations in the presence of interspecies interactions is the cumulative response of all individual bacteria. Due to random spatial distributions of the bacteria on the grid, some individuals in a population may be exposed to more interactions than others, causing heterogeneity of drug sensitivity within the populations. Comparing the distributions of the interaction target characteristics (i.e., MIC or maximal replication rate; Fig. 1) in populations exposed to different drug concentrations showed that in unstructured populations the average change in a population is of similar magnitude at above-MIC drug concentrations and varies slightly only for concentrations close to the MIC (Fig. 2). The observed similarity is most likely due to the large number of bacteria unaffected by interactions and the selected time point.

### Interaction and drug type determine how pharmacodynamics are affected by interspecies interactions

**Fig. 3:**
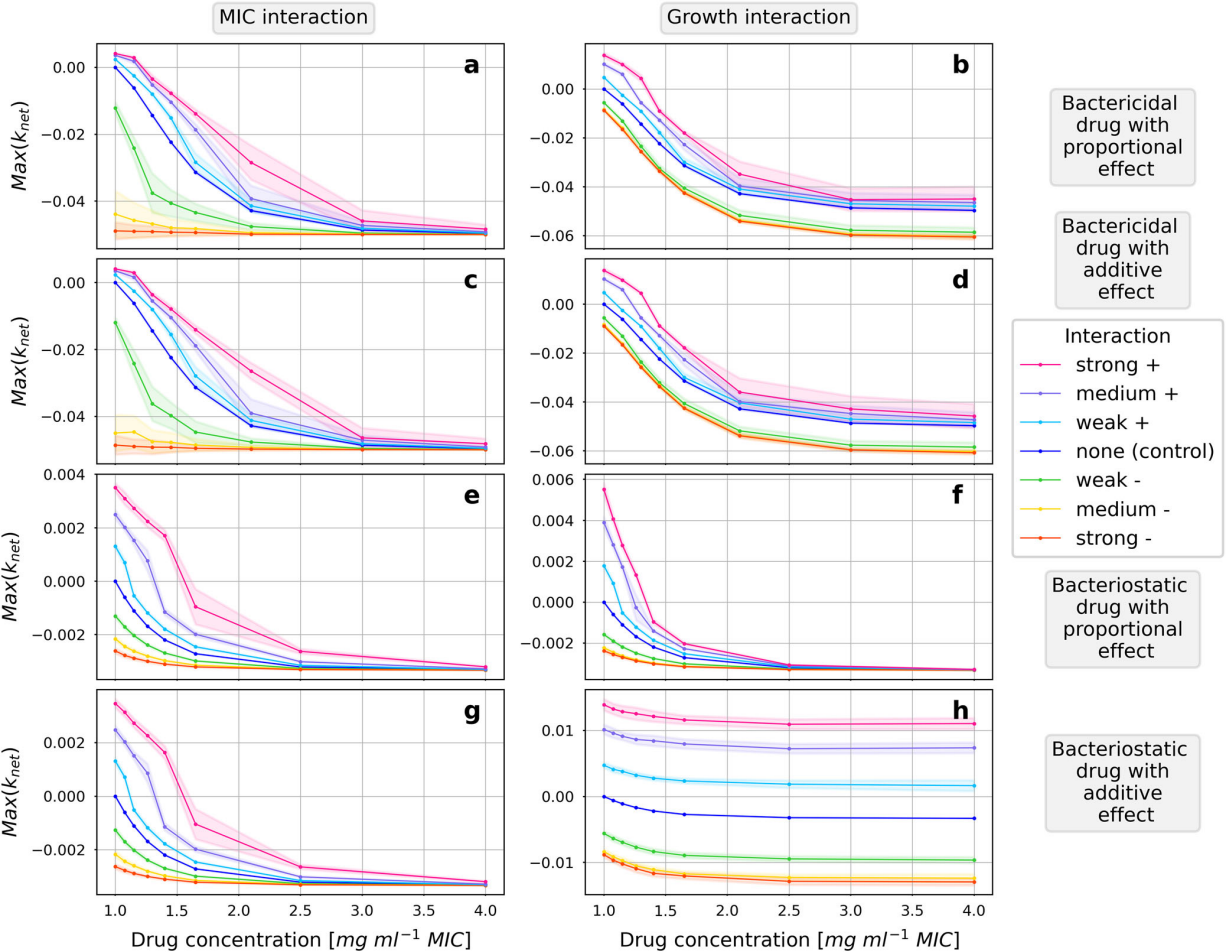
Interaction and drug type determine how pharmacodynamics are affected by interspecies interactions. The bacterial response of the focal pathogen to increasing drug concentrations is illustrated in concentration-response curves using the average maximal net growth rate of the focal pathogen population as a measure of response. The average maximal net growth at each concentration is the sum of the response of all individuals in the population averaged across 50 iterations of the simulation. The variation between iterations, measured as the standard deviation is indicated by the shaded area around the mean. In each subplot, bacterial response to one type of drug in the absence (dark blue line, the control) and in the presence of one type of interaction is illustrated. With increasing drug concentration, the net growth decreases in all cases whereby a negative value represents a dying population. At MIC concentration (*c* = 1), the net growth in the absence of interactions is zero as per definition for this model. The response of the pathogen to four types of drugs: a proportional, bactericidal drug, an additive, bactericidal drug, a proportional bacteriostatic drug and an additive bacteriostatic drug was tested. Two types of interactions which can either be positive or negative are included at weak, medium and strong strengths. The impact of interactions affecting the MIC of the focal pathogen, called MIC interactions, is illustrated in (**a**, **c**, **e**, **g**) and the impact of interactions affecting the maximal replication rate, called growth interactions, is summarized in (**b**, **d**, **f**, **h**). The strengths of the two types of interactions are chosen to be comparable at the x. concentration for the bactericidal, proportional, concentration-independent drug causing the same change in drug response in a perfectly mixed system which eliminates spatial effects. The strength of each type of interaction is the same for each drug allowing quantitative comparison between drugs.

We investigated the effect of interspecies interaction on pharmacodynamics of antimicrobials with five different pharmacological characteristics according to their concentration-response relationship (Table 1). The effect of interactions on pharmacodynamics, the interaction effect, was quantified separately for each tested drug concentration (see “Evaluation metrics” in the “Methods” section). We found that the presence of interspecies interactions can affect the antimicrobial concentration-response relationship in two ways: either the effect of interactions is present at all concentrations with similar effect size mimicking a vertical shift of the control curve, or it is only present at concentrations at which the drug effect is also sensitive to changes in drug concentration mimicking a horizontal shift (Fig. 3).

In the presence of interspecies interactions modulating growth, the pharmacological properties of the antimicrobials determined the type of effect observed in the concentration-effect relationship. The response to bactericidal drugs changed with a similar effect size at all tested concentrations compared to the control where no interactions are present (Fig. 3b, d). Interspecies interactions which negatively affect growth cause the pathogen to be more sensitive to the drug and lead to a decrease of the average maximal net growth rate. In contrast, positive growth interactions support the pathogen, causing it to be less sensitive, and lead to an increase of the average maximal net growth rate. With increasing strength of growth interactions, the observed interaction effect is larger for both negative and positive interactions. For bacteriostatic drugs, the response depends also on other pharmacological drug properties. The response to a proportional bacteriostatic drug is proportional to the drug effect only appearing in concentration ranges where the drug response is also sensitive to changes in the drug concentration (Fig. 3f). In contrast, the impact of growth interactions on the response to an additive bacteriostatic drug is similar as on bactericidal drugs (compare Fig. 3h to b, d).

**Fig. 4:**
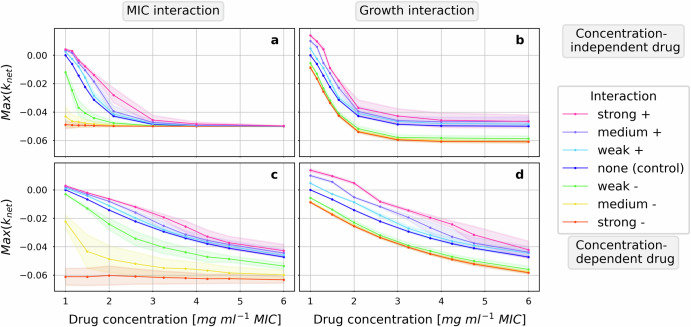
Growth interactions impact the response to concentration-dependent drugs also at high concentrations. The antibiotic sensitivity of the focal pathogen in the presence of interactions to two bactericidal drugs with proportional effect in a range of constant drug concentrations is illustrated in concentration-response curves. The two drugs differ in their chosen parameters for the Hill factor *n* and the maximal net growth rate at high concentrations *G*_min_ which define their concentration-dependency characteristic in the tested range of concentrations. While the concentration-independent drug (**a**, **b**, also Fig. 3a, b) reaches its maximal effect in the absence of interactions at approximately four times the MIC concentration, the concentration-dependent drug (**c**, **d**) does not reach its maximal effect within the range of tested concentrations and is therefore sensitive to concentration changes in the whole range. The impact of two types of interactions, the MIC interaction (**a**, **c**) and the growth interaction (**b**, **d**), are included at weak, medium and strong strengths.

Further, it is interesting to note that the change in population net growth rate caused by the negative growth interactions does not increase much with increasing interaction strength (Figs. 3b, d, f, h and 4b, d). The change in the net growth rate of the population is influenced by the interaction strength, the fraction of the population affected and how many interaction events occurred per bacteria. The impacts of the medium and strong interactions are very similar which is also reflected in the average change in the interaction target for the strong and medium negative growth interaction (Fig. 3e). This is caused by the choice of the interaction strength parameters and the natural limit for changes in replication rate and MIC, which is zero for both values. The natural limit of the replication rate is reached with a number of interactions than the natural limit of the MIC (compare Fig. 1g and e).

In contrast to growth interactions, MIC interactions impact bacterial response to all the drugs only in concentration ranges in which the drug effect is also sensitive to changes in drug concentration (Fig. 3a, c, e, g). The pharmacodynamics of concentration-dependent drugs is impacted in a similar fashion (Fig. 4). The simulated concentration-dependent drugs are formulated using the same equations differing from concentration-independent drugs only in the parameterization which explains the observed similarities (Table 1). However, unlike in the presence of concentration-independent drugs, the effect of the MIC interaction is also present at high concentrations of concentration-dependent drugs. For concentration-dependent drugs the drug effect has not reached its maximum at the upper limit of the tested concentration range and is thus still sensitive to changes in drug concentration.

This similarity between the population-averaged changes in MIC and the replication rate for all tested concentrations in unstructured populations (Fig. 2) allows for prediction of the type of effect based on the relation of the drug and the interaction target. Studying the mathematical equations of each drug (Eqs. (4a)–(9b)) and the relation between the parameter affected by the interaction and the drug concentration matches with the two types of observed effects, the horizontal and the vertical shift (Fig. 3). The equations of drugs affected vertically have an additive relationship between the affected parameter, the maximal replication rate *k*_rep,max_, and the drug concentration *c* which is in the drug effect *E* (compare Fig. 3b, d, h to Eqs. (4a), (4b), (4d) respectively). The equations of the drugs affected horizontally have a proportional relationship between the affected parameter, either the MIC or the *k*_rep,max_, and the drug concentration (compare Fig. 3a, c, e, f, g to Eqs. (9a), (9a), (9b), (4c), (9b) respectively).

Interestingly, the different types of qualitative effects, vertical or horizontal shifts, show differences in the variability of the population net growth between iterations represented as shaded areas around the mean (Figs. 3 and 4). Differences in the magnitude of the interaction effect are caused by differences in the spatial distribution of bacteria on the grid. The standard deviation is most pronounced for strong positive interactions (Fig. 3a–e, g) and negative interactions that cause a large change from the control (Figs. 3a, c and 4c) apart from growth interactions in the presence of a bacteriostatic drug (Fig. 3f, h). For those scenarios which show a vertical shift (Fig. 3b, d) in the presence of a bactericidal drug, the variability increases with increasing drug concentration.

Variability of the mean value between iterations is a feature of heterogeneous bacterial populations. The control populations without interactions are homogeneous and therefore do not show much variability between iterations. We hypothesize that larger heterogeneity of the interaction target within a population is associated with larger variability of the mean population value between iterations. This explains that stronger interactions show larger variability. It further explains the small amount of variability of the negative growth interactions (Fig. 3f, h). Due to the natural limit of the replication rate and the parameter choice, the target heterogeneity caused by negative growth interactions is small compared to negative MIC interactions (Fig. 1). Additionally, we would expect high drug concentrations to more strongly select in favor of heterogeneity when positive interactions are present and against heterogeneity when negative interactions are present. This could explain the increase in variability with increasing drug concentrations observed for some scenarios (Fig. 3b, d).

In conclusion, the effect of interspecies interactions on drug pharmacodynamics can be predicted qualitatively based on the drug effect formulation best describing a certain drug because the average change caused by interactions in a population is similar at above-MIC concentrations.

### Structured bacterial populations benefit more from positive interactions and are more resilient against negative interactions in the presence of bactericidal drugs

**Fig. 5:**
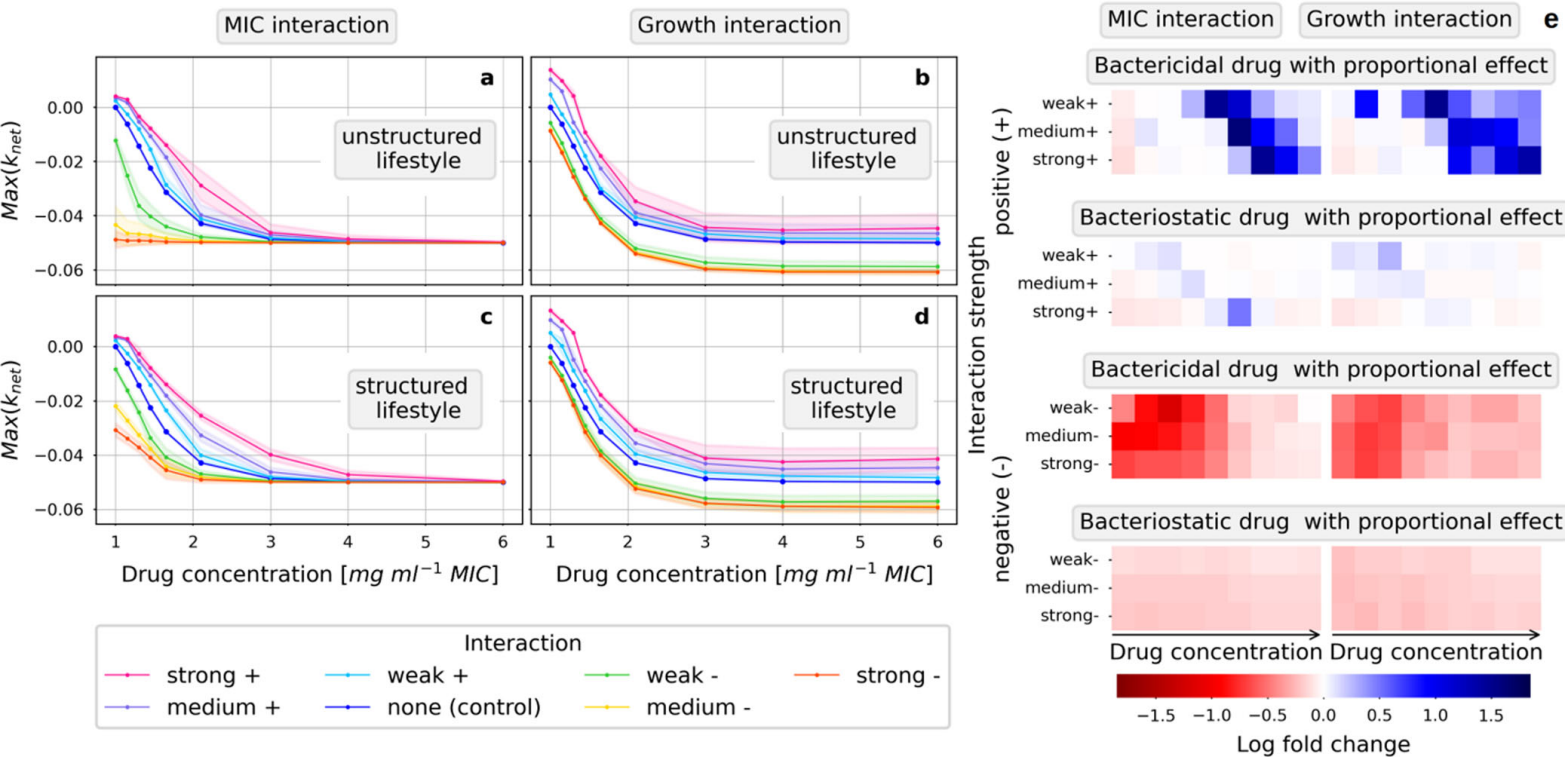
The effect of positive interactions on the response to bactericidal drugs is enhanced in structured populations compared to unstructured ones, while the effect of negative interactions is weakened. The focal pathogen’s sensitivity to a range of constant concentrations is tested in the presence of interactions with different lifestyle (structured or unstructured). In **a**–**d**, the response to a bactericidal, proportional, concentration-independent drug is presented in concentration-response curves using the maximal net growth as a measure of sensitivity. In **e**, a heatmap is presented which compares the effect size of the interaction impact on the concentration response curves in simulations in structured conditions to those in unstructured conditions using a log fold change measure for a bactericidal and a bacteriostatic drug. In unstructured conditions (**a**, **b**) the bacteria each move one step each timepoint which causes a high amount of mixing of the two populations. In contrast, in structured conditions (**c**, **d**), the bacteria do not move. The presence of interactions, either the MIC interaction (**a**, **c**, **e** left column) or the growth interactions (**b**, **d**; **e** right column), effect the response differently in the different lifestyle conditions (compare **c** to **a** and **d** to **b**). The log fold changes of the relative interaction effect of negative (**e** the lower half) and positive (**e** the upper half) MIC and growth interactions for two concentration-independent drugs are summarized in a heatmap. A positive log fold, in blue, shows that the interaction effect in the scenario in structured condition is larger than in unstructured conditions, while a negative log fold change, in red, indicates a smaller effect. The log fold change values are calculated for each concentration by comparing the relative interaction effect in structured condition to the relative interaction effect in unstructured condition. We observe that for positive interactions, the relative interaction effects are all positive, whereas they are all negative for the negative interactions.

We investigated the impact of bacterial lifestyle (structured and unstructured) on the magnitude of the interaction effect and found that in the presence of bactericidal drugs, bacteria benefit more from positive interactions and are more resilient against negative interactions in structured populations than in unstructured ones (Fig. 5). While the bacterial movement in unstructured populations is undirected, the speed of movement is an important factor determining the amount of mixing of the two species and therefore the number of possible interactions between the species. In silico bacterial populations with fast moving bacteria represent well-mixed planktonic populations, called unstructured populations. Populations without movement represent very dense populations, biofilm populations or populations living in an environment that restricts movement and are called structured populations.

Comparing concentration-response curves of unstructured populations to concentration-response curves of structured populations showed differences in how strongly the interactions affect the bacterial sensitivity (Fig. 5a–d comparing the upper to the lower row). Visual analysis of the example for a bactericidal drug suggests that negative interactions of either type decrease the maximal net growth at certain concentrations less in structured conditions compared to unstructured ones. This shows that the bacterial population is less sensitive to the drug when the bacteria mix less because the impact of negative interactions is weaker. Further, we observed that positive interactions are affected in the opposite direction. In structured conditions positive interactions increase the maximal net growth at certain concentrations more than in unstructured conditions pointing towards an enhanced effect of the presence of positive interactions.

The magnitude of the interaction effect depends on the number of individual interactions occurring which is determined by the distribution of the two species on the grid. A spatial distribution that reduces the number of possible interactions will hamper the interaction’s overall effect; a spatial distribution that maximizes the number of interactions will enhance the effect of the interactions. In the beginning of each simulation, individuals from each species are placed randomly on the grid. This distribution then changes due to selective pressure of the drug and, in the case of unstructured populations, the movement of bacteria.

Quantitative comparison of the interaction effect in structured and unstructured conditions showed that structured conditions and the presence of a bactericidal drug enables the species distribution to change to the benefit of the bacteria over time by enhancing the effect of positive and weakening the effect of negative interactions (Fig. 5e). To quantitatively compare the differences between concentration-response curves in structured and unstructured conditions, the magnitude of the interaction effect relative to the control, the relative interaction effect (*ρ*), was determined for each concentration. It describes the percentual change in response of bacteria to the drug in the presence of interactions versus in their absence. Relative interaction effects in structured and unstructured conditions were then compared to each other using a log fold change. Whereby a positive log fold change indicates that the interaction effect is stronger in structured conditions compared to unstructured ones, and a negative log fold change indicates that the effect is weaker.

The response to bactericidal drugs is affected by the bacterial lifestyle (structured or unstructured) for both positive and negative MIC and growth interactions (Fig. 5e). The response to bacteriostatic drugs is unaffected except for the additive bacteriostatic drug. There are only minor differences between the proportional and additive drugs in the different lifestyle conditions except for the negative growth interaction for the additive bacteriostatic drug. Interestingly, a structured lifestyle is more beneficial for survival of the bacterial population in the presence of any type of interaction and any bactericidal drug; it enhances the effect of positive interactions and weakens the effect of negative interactions confirming the initial observation from Fig. 5a–d. Further, the concentration at which the largest difference was observed shifts to a higher concentration with increasing strengths of positive interactions and to a lower concentration with increasing strengths of negative interactions. However, in contrast to expectation, the log fold change for stronger interactions is not bigger than for weak interactions.

The differences observed for bactericidal and bacteriostatic drug can be explained by the different types of selection pressures they exhibit. When bactericidal drugs are present, bacteria that are in an unfavorable position, either receiving too few positive or too many negative interactions, are killed at a higher rate than those in more favorable positions. This creates a more optimal distribution of the species on the spatial grid at the end of a timestep than at the beginning. In structured populations, this distribution is conserved and in the next timestep few bacteria are likely to die because many of them are already in favorable positions overall benefiting the population by either maximizing or minimizing the number of individual interactions in the timestep. Bacteriostatic drugs however do only inhibit growth but not kill bacteria hence they have a much weaker selective effect. Therefore, bacteria in unfavorable positions will still survive and suffer again in the next timestep, cannot grow and then eventually die from natural causes in the presence of bacteriostatic drugs.

### Long-range interactions can be more harmful to bacteria because they cannot benefit as much from the spatial rearrangement

**Fig. 6:**
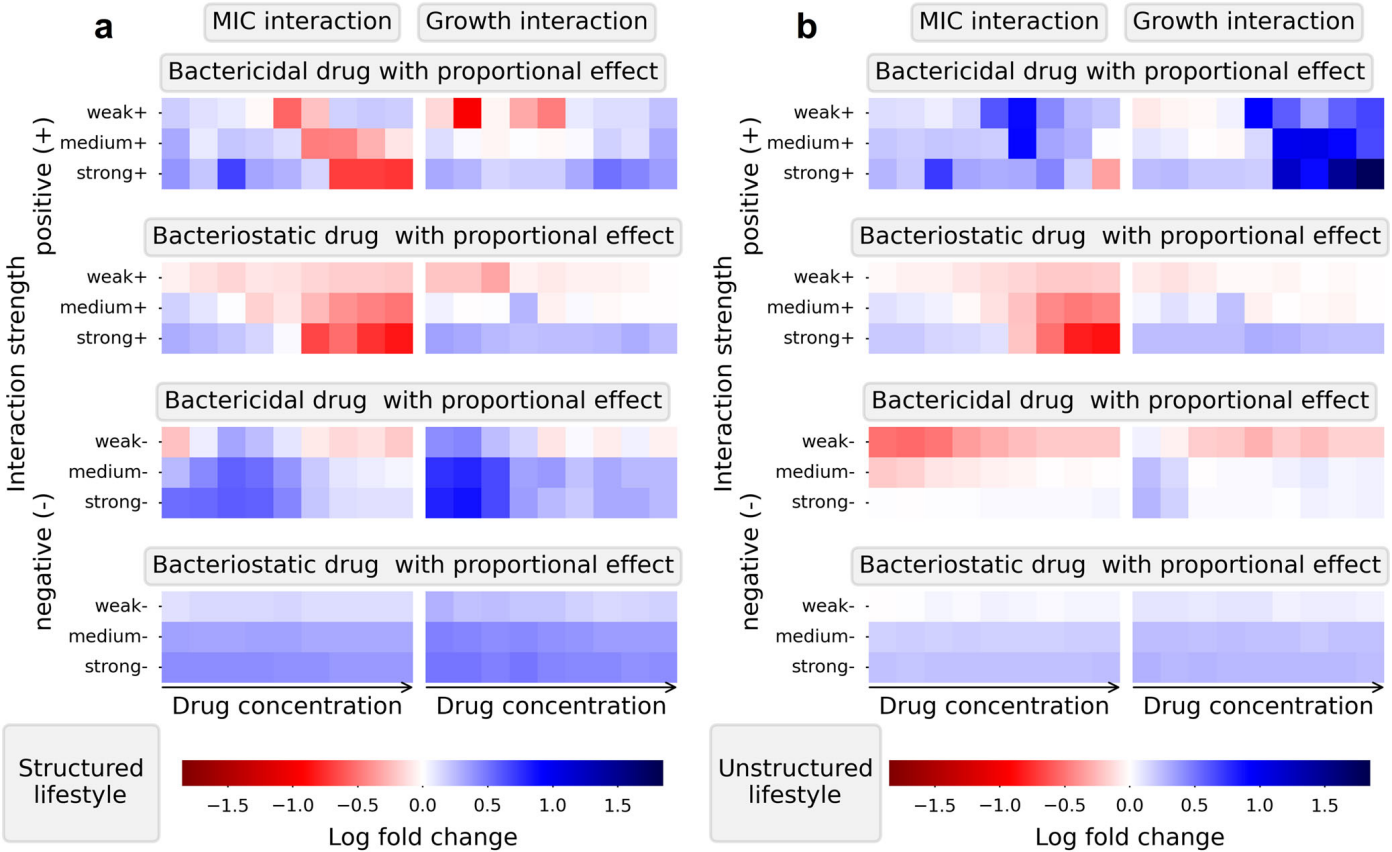
Long-range interactions are more harmful to bacteria because they cannot benefit as much from the spatial rearrangement to avoid or benefit from interactions. The log fold change compares the response of bacteria in presence of long-range interactions to short range ones. Simulations were performed in unstructured (**b**) and structured conditions (**a**). A positive log fold, in blue, shows that the impact of long-range interactions on the bacterial sensitivity is stronger than the impact of short-range interactions.

We investigated the impact of the interaction distance on the magnitude of the interaction effect and found that long-range interactions can be more harmful to bacteria because they reduce the chance for an altered spatial distribution to appear (Fig. 6). The interaction distance is a crucial factor determining which species distribution is beneficial therefore influencing adaptation of the bacterial population and the magnitude of the interaction effect.

We considered a longer interaction distance of up to 200 μm because this leads to overlapping interaction groups compared to a short distance and is expected to affect spatial distribution. Importantly, the maximal distance of an interaction determines how many individuals are within a bacterium’s radius of interaction and how many individual interactions occur. A larger interaction radius, caused by long-range interactions, will include more bacteria than a smaller one. To investigate the effect of the interaction distance on the magnitude of the interaction effect, we corrected for the higher number of expected interactions in a perfectly mixed system when simulations are performed by adapting the interaction strengths (see “Interspecies interactions” in the “Methods” section). The observed differences of the magnitude of the interaction effect are thus only caused by the influence of the different interaction distances on the species distribution on the grid and not by differences in overall interaction strengths.

Comparing the effect of long-range interactions to short-range interactions when the bacteria are not moving reveals that a longer interaction distance can have an enhancing (indicated in blue), a weakening (in red) or no effect (in gray) depending on the type of interaction (Fig. 6a). We observed that at certain concentrations, positive long-range MIC interactions cause less of a sensitivity change than positive short-range ones at the same strength. Additionally, we found that negative long-range interactions are slightly stronger at medium and strong interaction strengths than short-range ones. This can be explained because a longer interaction distance decreases the chance for an interaction group with a species composition that optimizes the number of interactions to occur. The reason that a longer interaction distance hinders optimal spatial rearrangement is that the interaction groups are larger hence more average and that they overlap. In conclusion, in the presence of long-range interactions, bacteria have less chance to adjust to the presence of the interactions and drugs.

When bacteria are moving, the effect of positive long-range interaction on the response to bactericidal drugs is enhanced at some concentrations (illustrated in blue in Fig. 6b). The most affected concentration increases with increasing interaction strengths for the growth interaction. Further, a long interaction distance weakens the effect of weak negative interactions on the response to bactericidal drugs slightly (indicated in red), however it does not affect strong negative interactions. We also observed that medium and strong MIC interactions affect the response to bacteriostatic drugs less strongly when the interactions have a longer interaction distance.

Overall, the observed pattern in Fig. 6b shows similarities to both Figs. 5e and 6a which can be explained: Because of the longer interaction distance, the fraction of previous timestep neighbors which are also neighbors in the following timestep is increased compared to a short distance. This is also the case when comparing structured to unstructured conditions. In other words, when the interaction distance is longer, the bacterial population appears more static.

## Discussion

We have developed a modeling framework which describes the dynamical response of multispecies bacterial communities to antimicrobials. Our analysis demonstrates that both the type of interaction and the type of drug determine how the pharmacodynamics of the focal pathogen are impacted and that this can be predicted qualitatively (Figs. 3 and 2). Furthermore, we found that changes in the bacterial lifestyle (structured or unstructured) and the interaction distance can influence the magnitude of the impact of interactions highlighting the importance of spatial organization of the species. The magnitude of the interaction impact is altered to the benefit of the bacterial population when the bacterial lifestyle, interaction distance and drug characteristics allow for adaptation of the bacterial population by changing its spatial distribution over time. In structured conditions, the bacterial population benefits more from beneficial interactions in the presence of bactericidal drugs and is more resilient against harmful interactions (Fig. 5). Similarly, when interactions have a longer interaction distance, beneficial interactions affecting the bacteria’s susceptibility are less beneficial while harmful interactions of any type are more harmful in the presence of any type of drug than when they have a short interaction distance (Fig. 6).

This works demonstrates that the effect of interactions can be predicted based on the mechanism of action of the drug in relation to the target of interaction (Fig. 3). This prediction is possible because the population-averaged changes of the MIC and the replication rate at the time of maximal net growth are similar for all tested concentrations (Fig. 2). This provides information of which type of antimicrobial may be able to benefit from or avoid the effects of interspecies interaction (Fig. 3). When an interaction affecting growth is dominant, the mechanism of action of a drug should be considered. At high concentrations, a bacteriostatic drug that acts proportionally to the rate of replication could be considered when the effect of interactions are to be avoided, while a bactericidal drug could be considered when the interactions are competitive and the treatment might benefit from the interaction effects. When interactions affecting resistance are dominant, we suggest that the concentration dependency of a drug should be considered when designing treatments (Fig. 4).

Our findings show that the bacterial lifestyle and the interaction distance can influence the magnitude of the impact of interactions highlighting the importance of spatial organization of the species. Previously, interspecies interactions have been shown to be a main driver of spatial organization of bacterial species^33,34^. Further, the importance of spatial organization of bacterial species for community function, both in the absence^19,20^ and in the presence of antimicrobial treatment^31,35–37^ has been demonstrated. In the absence of antimicrobial drugs, it has been found that the amount of mixing between two species and the interaction distance are crucial for community function and growth in a multispecies cross-feeding community^19^. Conversely, in the presence of antimicrobials, it has been shown that antimicrobial exposure of a multispecies biofilm causes a spatial restructure of the community which promotes interspecies interactions protecting the bacteria against imipenem^36^. Similarly, it has been shown that disturbances of the spatial organization in a biofilm can disrupt beneficial communication between bacterial species which in consequence decreases their expression of virulence factors^35^. In comparing our findings with biofilm studies, it is essential to note that our model captures certain aspects of biofilm populations, particularly the restricted movement of individual bacteria within structured communities. However, it does not account for other characteristics that may affect the antibiotic sensitivity of the biofilm community. Overall, these findings highlight the potential of interventions targeting the spatial organization for antimicrobial treatment of PMIs and are in agreement with our findings.

Our analysis highlights the potential of additional therapeutics that target the bacterial lifestyle, interaction distance and the spatial organization of a multispecies community. More mixing in the population could decrease the effects of beneficial interactions between species and enhance harmful ones in the presence of bactericidal drugs (Fig. 5). Similarly, in structured microbial populations, an increase in the interaction distance could alter the magnitude of the effect of resistance interactions to the detriment of the bacteria because it reduces the chance that an optimal spatial arrangement develops (Fig. 6).

Our modeling framework for PMIs has an emphasis on interactions between individual bacteria and associated population heterogeneity. Our model offers a distinctive advantage by combining rule-based modeling techniques with deterministic ODE modeling. We used ODEs to describe the growth and kill rates of bacteria, while ABM techniques were employed to represent bacterial movement and interactions. The use of a stochastic approach such as the ABM facilitated the simulation of a heterogeneous population and interactions between individual bacteria in a straightforward manner. By incorporating ODEs, we limited the variability between iterations of the model solely to the variability arising from interspecies interactions which allows for easier interpretation of the simulation output and offered a clearer understanding of the impact of these interactions.

The developed model framework can be used to simulate pairwise or higher-order interaction networks within bacterial populations comprising multiple species in the presence of hypothetical antimicrobial drugs. Additionally, our model allows for the integration of pharmacokinetic models enabling the representation of temporal variations in drug concentrations. Moreover, the framework is readily extendable with other types of interactions, more detailed interaction mechanisms, multiple species, spatial heterogeneity of drug concentrations, such as drug gradients, and the action of immune cells. This adaptability opens up avenues for investigating a wide range of research questions related to polymicrobial populations and their response to antimicrobial treatment. One research area where our model holds promise is the study of pharmacological perturbations of microbiomes^38,39^. By utilizing our framework, researchers can explore the effects of various pharmacological treatments on the dynamics and composition of microbiomes.

While our model’s design effectively supports the systematic analysis of interspecies interactions, it is challenging to compare our findings directly to experimental studies. This limitation arises partly from our deliberate decision to maintain a general approach and partly from the design of our in silico experiments. The scarcity of in vitro experiments focusing on PMIs restricts the experimental setup and measuring techniques available. Certain design elements in our simulations, which enhance result interpretability, cannot be replicated in an experiment. For instance, co-culturing two species under varied drug concentrations while maintaining a constant number of one bacterium is difficult to achieve experimentally. Our study aims to provide hypotheses, which can serve as a valuable starting point for future experimental research.

Designing experiments to validate the results derived in this study presents significant challenges. Key considerations include the selection of suitable bacterial strains based on their drug sensitivity, motility and specifically characterized interaction mechanisms. Further, the choice of measuring techniques is critical; the use of fluorescently labeled strains in coculture experiments at a single cell or population-level experiments will be essential. Depending on the experimental focus and questions, different approaches may be employed. Population-level experiments, aiming to assess outcomes such as drug response curves, may utilize co-culture assays to measure changes in total fluorescent signal of strains. Conversely, experiments examining spatial dynamics may employ imaging techniques to quantify in time spatial distribution and clustering of cell types in the presence of multiple drug concentrations. We believe that the model framework developed in this manuscript can provide valuable guidance for designing targeted experiments investigating the pharmacodynamics of polymicrobial infections, potentially reducing experimental effort.

The interpretation of our results is constrained by the simplified representation of interspecies interactions in our model and the choice of interaction parameters. Rather than considering the specific biochemical mechanisms underlying these interactions, our model focuses on their overall outcome. While most interaction parameters were set within a realistic range, determining the precise values of interaction strength and interaction distance from the literature was challenging. The mechanism of the interaction could provide important information for the parameterization of the interaction strength and the interaction distance. However, comprehensive information regarding the mechanism and quantitative properties of many interspecies interactions influencing drug sensitivity is currently lacking. As a result, we assigned them based on realistic population-level outcomes.

It is important to acknowledge that our study primarily explores unidirectional, pairwise interactions and assumes that one species is insensitive to drug treatment, even though complex higher-order, multi-directional interaction networks are likely to be present in PMIs. Our aim was to systematically analyze the impact of interspecies interactions on drug pharmacodynamics; thus we focused on the effect of interactions on one bacterial pathogen. It is important to point out that this assumption limits our ability to fully capture the intricate dynamics within PMIs. However, it is worth noting that our developed modeling framework is versatile and can be used to simulate bidirectional and higher-order interaction networks. We believe that our chosen approach, which unveils individual interaction dynamics, can contribute to shedding light on the intricate dynamics of higher-order interaction networks.

In summary, our study presents a comprehensive analysis of the impact of interspecies interactions on bacterial pharmacodynamics. The developed model is a versatile framework which can be easily adapted for a wide range of research questions related to polymicrobial populations and their response to pharmacological perturbations. Our findings offer insights into selecting antimicrobial drugs that can benefit from or avoid the effects of interspecies interactions and highlight the potential of interventions targeting spatial organization. Moreover, we provide hypotheses which can be a valuable starting point for future experimental research and may pave a way for the development of targeted treatment strategies that consider the complex dynamics of these infections, ultimately improving patient outcomes.

## Methods

### Model

We developed an integrated hybrid modeling framework that describes a microbial community consisting of multiple species exposed to antimicrobial drugs by combining an ABM approach and ODEs. An ABM approach was used to describe movement of bacteria and interactions between them, whereas ODEs were used to describe the growth of the bacteria and the effect of antimicrobial drugs. In the model, each bacterial cell in the population is represented by a computational agent, which we will refer to as bacteria in the remainder of this manuscript. The bacteria are positioned on patches of a two-dimensional grid with periodic boundary conditions representing an infection volume of approximately 1 nanolitre which can hold up to 1000 bacteria (Table 2 “Grid and movement”). The grid acts as a simplified representation of space providing information about which individual bacteria are in proximity to interact. During the simulation, bacteria can move on the grid in an undirected manner which represents passive diffusion.

**Fig. 7:**
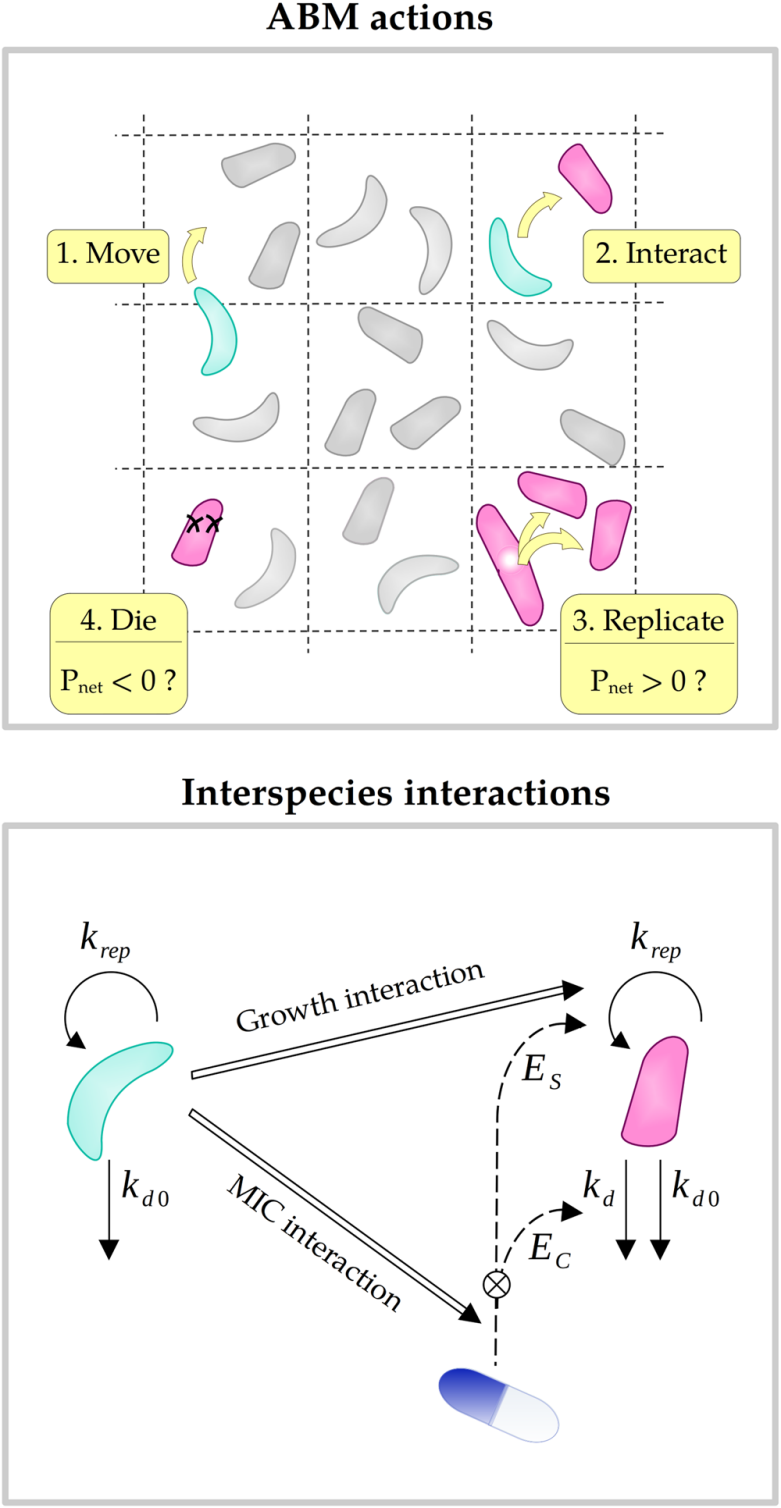
An agent-based modeling framework of interspecies interactions in polymicrobial infections. Individual bacteria are represented by computational agents which are placed on a spatial grid. At every timestep, bacteria can perform four actions: move, interact, replicate (*k*_rep_) and die as a result of natural causes (*k*_*d*0_) or drug treatment (*k*_*d*_). Two types of interaction between the bacterial species are incorporated which can either affect their replication rate or MIC. Furthermore, the model includes different types of drugs that inhibit bacterial replication (*E*_*s*_) or kill bacteria (*E*_*c*_).

Simulations of the model were performed in discrete, consecutive time steps. At every time step, each bacterium is activated and can perform four actions in a fixed order: it can move to a neighboring patch on the grid, interact with other bacteria, replicate or die as a result of natural causes or drug treatment (Fig. 7). Upon activation of a bacterium, each of the actions occurs with a predefined probability. First, the bacterium moves with probability *p*_move_ = *n*_steps_. Second, the bacterium interacts with each bacterium of another species that is currently in its interaction distance with probability *p*_interact_ = 1. Third, the bacterium replicates or dies with probability ∣*p*_net_∣ whereby positive values of *p*_net_ indicate a chance to replicate and negative values a chance to die. The value of *p*_net_ is individual to every bacterium and can be altered by interactions. It is derived from the deterministic population net growth rate *k*_net_ and the length of the simulated timesteps Δ*t* according to:

1 $${p}_{{\rm{net}}}={k}_{{\rm{net}}}\Delta t.$$

As a result, the dynamics of a bacterial population without interspecies interactions simulated in the ABM framework closely approximates deterministic dynamics described by ODEs (Eqs. (3)–(9b)).

#### Interspecies interactions

The model incorporates two types of interspecies interactions which represent interactions that affect individual bacteria’s sensitivity to antimicrobials that can occur when bacteria are in close proximity. The description of the interactions focuses on the outcome of the interactions, a change in different interaction targets *T* ∈ {*k*_rep,max_, MIC}, but does not specify their mechanism of action. The growth interaction targets the maximal replication rate *k*_rep,max_, while the MIC interaction targets the Minimum Inhibitory Concentration (MIC) of an individual bacterium. Each type of interaction can negatively or positively alter the interaction target of the affected bacterium, respectively called positive and negative interactions. In this project, the MIC was used as a measure of resistance of individual bacteria. The MIC of a population is the average of all individual bacteria’s MIC values which approximately corresponds to the experimentally used MIC measure signifying zero visible net growth after 24 h.

Interspecies interactions are characterized by their target *T*, their strength and the maximal distance at which they can take place. Interactions can be weak, medium or strong and occur at short or long range. During simulation, interactions occur between an activated bacterium and all bacteria of another species within interaction distance. Each interaction alters the initial value of the interaction target *T*_0_ by a fixed fraction *f* according to its strength. While positive interactions cause an increase, negative interactions cause a decrease. When a bacterium is affected by multiple interactions in one timestep, the fractional changes are summed up for all *N* interactions to derive the altered value of the interaction target *T*_Δ_ of the bacterium according to:

2 $${T}_{\Delta }={T}_{0}\left(1+\mathop{\sum }\limits_{i=0}^{N}f\right)\,\,\text{for}\,\,T\in \{{k}_{{\rm{rep}},{\rm{max}}},{\rm{MIC}}\}.$$

The effect of an interaction is only present in the timestep in which the interaction occurs and is not inherited by daughter cells upon replication.

The parameter values of fraction *f* of MIC interactions, which define the strength of an interaction, were chosen so that the resulting changes in MIC range up to a four-fold change for a strong interaction. Fraction values of the growth interactions were matched to the MIC interaction for each strength to ensure compatibility between these interaction types. The change in the maximal net growth rate of a population in the reference conditions is the same whether a growth or an MIC interaction is present. The reference conditions are a unstructured population (with fast movement) interacting at short-ranges in the presence of a proportional, bactericidal, concentration-independent drug at a certain concentration.

The interaction strengths of long-range interactions were corrected to ensure that the overall effect is equal to the effect of short-range interactions under reference conditions. Thus, the fraction value *f* of a long-range interaction and its maximal value were normalized to the higher number of interactions in a well-mixed population. As a result, the observed differences in the magnitude of the interaction effect when comparing interactions with different interaction distances under conditions other than the reference conditions are caused by the influence of the different interaction distances on the species distribution on the grid and not by differences in the number of interactions.

The interaction distances for short- and long-range interactions were defined based on the number of bacteria which form an interaction group (Table 2 “Interactions”). When a bacterial population is growing at capacity in our simulations, an average interaction group of a short-range interaction encompasses 6.8 bacteria and half at the start of each simulation. This is approximately in line with experimental measurements reporting interaction groups consisting of 3 to a few dozen bacteria for short-range interactions^20^. It is worth mentioning that the interaction distance measured in the same set of experiments is not comparable because of differences in the assumed bacterial density compared to our model. In the experimental setup bacteria are fixated in a microfluidic device in a very dense distribution. However, our in silico model allows for movement of bacteria, and we have assumed a lower maximal bacterial density based on polymicrobial lung infections of patients with cystic fibrosis^40^ (Table 2). Further, we assumed an interaction distance that is twice as long and encompasses 9 times as many bacteria could be considered long-range compared to short-range ones.

#### Bacterial growth and drug effect

The net growth dynamics of the bacterial population exposed to antimicrobials in the absence of interspecies interactions were described by ODEs that were integrated in the ABM framework and summarized below. The growth of a bacterial population *B* consisting of multiple species is described by:

3 $$\frac{dB}{dt}={k}_{{\rm{net}}}B.$$

with the net growth rate *k*_net_

$${k}_{{\rm{net}},x}=\left\{\begin{array}{ll}{k}_{{\rm{rep}},\Delta }-{k}_{d0}(1+{E}_{c,p})\quad &\,{\text{for}}\, {\text{x}}\,=\{\,\text{c,p}\,\}\quad (4{\rm{a}})\\ {k}_{{\rm{rep}},\Delta }-{k}_{d0}-{E}_{c,a}\quad &\,{\text{for}}\, {\text{x}}\,=\{\,\text{c,a}\,\}\quad (4{\rm{b}})\\ {k}_{{\rm{rep}},\Delta }(1-{E}_{s,p})-{k}_{d0}\quad &\,{\text{for}}\, {\text{x}}\,=\{\,\text{s,p}\,\}\quad (4{\rm{c}})\\ {k}_{{\rm{rep}},\Delta }-{E}_{s,a}-{k}_{d0}\quad &\,{\text{for}}\, {\text{x}}\,=\{\,\text{s,a}\,\}\quad (4{\rm{d}})\end{array}\right.$$

determined from the replication rate *k*_rep_, the natural death rate *k*_*d*0_ and the drug effects *E*_*x*_ ∈ {*E*_*c*,*p*_, *E*_*c*,*a*_, *E*_*s*,*p*_, *E*_*s*,*a*_} which are incorporated in four different ways representing various types of antimicrobials (Table 1). The effect of bacteri**c**idal antimicrobials which kill bacteria, is applied to the natural death rate either in a proportional or an additive fashion and which are denoted by *E*_c,p_ and *E*_c,a_, respectively. Similarly, to describe the effect of bacterio**s**tatic antimicrobials which inhibit bacterial growth, the effect is applied to the replication rate in a proportional or an additive fashion and respectively denoted by *E*_s,p_ and *E*_s,a_. We chose these four semi-mechanistic equations (Eqs. (4a)–(4d)) because they have empirically been found to describe the drug pharmacodynamics of various antimicrobial drugs correctly^41,42^.

The total number of bacteria is limited by the carrying capacity *K* reducing the replication rate:

5 $${k}_{{\rm{rep}},0}={k}_{{\rm{rep}},{\rm{max}},0}\left(1-\frac{B}{K}\right)$$

at high numbers of bacteria. The replication rate altered by growth interactions is denoted by *k*_rep, Δ_ and similarly described as:

6 $${k}_{{\rm{rep}},\Delta }={k}_{{\rm{rep}},{\rm{max}},\Delta }\left(1-\frac{B}{K}\right).$$

The relationship between the drug effect *E*_*x*_ and the drug concentration *C* is assumed to be sigmoidal:

7 $${E}_{x}(C)={E}_{{\rm{max}},x}\frac{{C}^{h}}{{C}^{h}+{EC5{0}_{x}}^{h}}$$

with the Hill factor *h*, the maximal effect *E*_max,*x*_:

8 $${E}_{{\rm{max}},x}=\left\{\begin{array}{ll}\frac{{k}_{{\rm{rep}},0}-{G}_{{\rm{min}}}}{{k}_{d0}}-1\quad &\,{\text{for}}\, {\text{x}}\,=\{\,\text{c,p}\,\}\\ {k}_{{\rm{rep}},0}-{k}_{d0}-{G}_{{\rm{min}}}\quad &\,{\text{for}}\, {\text{x}}\,=\{\,\text{c,a}\,\}\\ 1\quad &\,{\text{for}}\, {\text{x}}\,=\{\,\text{s,p}\,\}\\ {k}_{{\rm{rep}},0}\quad &\,{\text{for}}\, {\text{x}}\,=\{\,\text{s,a}\,\}\end{array}\right.$$

and the drug concentration for which the effect is half-maximal *EC*50_*x*_:

$$EC5{0}_{x}^{h}=\left\{\begin{array}{ll}{{\rm{MIC}}_{\Delta }}^{h}\frac{{G}_{{\rm{min}}}}{{k}_{d0}-{k}_{{\rm{rep}},0}}\quad &\,{\text{for}}\, {\text{x}}\,=\{\,\text{c,p/a}\,\}\,\quad (9{\rm{a}})\\ {{\rm{MIC}}_{\Delta }}^{h}\frac{{k}_{d0}}{{k}_{{\rm{rep}},0}-{k}_{d0}}\quad &\,{\text{for}}\,{\text{x}}\,=\{\,\text{s,p/a}\,\}.\quad (9{\rm{b}})\end{array}\right.$$

The relations of *E*_max,*x*_ and *EC*50_*x*_ have been derived analytically, similar to ref. ^43^, from assumptions about the net growth rate at very high:

10 $$\mathop{\lim }\nolimits_{c\to \infty }(\psi (c))=\left\{\begin{array}{ll}{G}_{{\rm{min}}}\quad &\,{\text{for}}\, {\text{x}}\,=\{{\rm{c}},{\rm{p}}/{\rm{a}}\}\\ -{k}_{d0}\quad &\,{\text{for}}\, {\text{x}}\,=\{{\rm{s}},{\rm{p}}/{\rm{a}}\}\end{array}\right.$$

as well as at MIC concentration. It is assumed that the net growth is zero when concentration equals the MIC concentration:

11 $$c={\rm{MIC}}:\psi ({\rm{MIC}})=0.$$

Whereby *G*_min_ denotes the minimal net growth rate reached at very high concentrations of bactericidal drugs (for x = {c,p/a}).

### Simulation design

Bacterial populations consisting of two bacterial species which can interact unidirectionally were simulated in the presence of antimicrobial drugs (for simulation settings see Table 2). Antimicrobial mono-treatment was simulated at constant concentrations ranging from a concentration equal to the MIC to 6 times its value for each of the hypothetical drugs (Table 1). Fifty iterations of the simulation were performed for each type of interaction, interaction strength, interaction distance, movement speed, drug type and drug concentration.

**Table 1:** Simulation of antimicrobial drug treatment

Pharmacological properties	Equations	Parameters		Simulated concentrations
		*h* [−]	*G*_min_ [min^−1^]	[mg ml^−1^ MIC]
Bactericidal drug with proportional effect	Eq. (4a)			
concentration-independent		5	−0.05	1, 1.15, 1.3, 1.45, 1.65, 2.1, 3, 4, 6
concentration-dependent		1.75	−0.0654	1, 1.5, 2, 2.6, 3.25, 3.75, 4.25, 4.75, 6
Bactericidal drug with additive effect	Eq. (4b)	5	−0.05	1, 1.15, 1.3, 1.45, 1.65, 2.1, 3, 4, 6
Bacteriostatic drug with proportional effect	Eq. (4c)	4	–	1, 1.075, 1.15, 1.26, 1.4, 1.65, 2.5, 4, 6
Bacteriostatic drug with additive effect	Eq. (4d)			

Hypothetical antimicrobial drugs with distinct pharmacological properties are defined. They are described by four equations Eqs. (4a)–(4d) and their respective parameters, the Hill coefficient *h* and the maximal net growth rate *G*_min_. For one of the drug equations (Eq. (4a)), two sets of parameters were chosen which represent a concentration-independent and a concentration-dependent version. The chosen parameter sets for all other drugs represent concentration-independent versions. Parameters were chosen to fulfill rules of thumb describing the characteristics of antibiotics classified as concentration-(in)dependent according to ref. ^41^. With the chosen parameter set, the hypothetical concentration-independent drugs reach their maximal effect at approximately four times a concentration equal to the MIC which is common for concentration-independent drugs^41^. The parameter set of the hypothetical concentration-dependent drug was chosen so that it reaches approximately eighty percent of their maximal effect within the clinical range which is assumed to range until six times a concentration equal to the MIC^41^.

Simulations of the model with different parameters for the movement, representing bacterial lifestyle, and the interaction distance were performed. An unstructured lifestyle, which is indicated by movement of the bacteria at every timestep, represents a planktonic population. Planktonic bacteria are not attached to each other or any surface and are freely floating and moving due to diffusion. The model is also simulated without movement (*n*_steps_ = 0) which represents structured bacterial population with severely restricted movement such as biofilms or very dense populations. We chose two settings for the interaction distance which represent short- and long-range interactions.

Each simulation run was initialized by placing in total 500 bacteria with equal amount of each species randomly on the grid (Table 1). During a simulation, bacteria belonging to the species that initiate interactions are insensitive to drug treatment. As a result, the number of bacteria from this species stays constant, which allows us to systematically investigate how the focal bacterial species is affected by the interactions and the drug. At the end of every timestep *t* of the simulation run, the following data were recorded: the number of bacteria *B* of each species *j* (*B*_*j*_), the probability to divide or die (*p*_net,*i*,*t*_), the maximal replication rate (*k*_rep,max,Δ,*i*_) and the MIC_*i*_ of every living bacterium *i*.

### Evaluation metrics

The drug response of the focal pathogen population was measured by the maximal absolute value of the population net growth rate *k*_net_ during the runtime of one iteration. The maximal net growth rate of a bacterial population is a commonly used measure of the antimicrobial effect because it is a direct measure of fitness^44^. It was determined from the average stochastic net growth rate *p*_net_ of every bacterium *i* at time *t* as follows:

12 $${k}_{{\rm{net}},{\rm{max}}}:= \mathop{\max }\limits_{t}({k}_{{\rm{net}}})=\mathop{\max }\limits_{t}(\overline{{p}_{{\rm{net}},i,t}})$$

and is denoted by *k*_net,max_. The drug response to different drug concentrations was illustrated for each simulated scenario in a concentration-response curve.

The effect of the interactions on the drug response of the focal pathogen was quantified for every drug concentration to compare the simulated scenarios to each other and called the relative interaction effect *ρ*:

13 $$\rho =\frac{{{k}_{{\rm{net}},{\rm{max}},\Delta }}^{c = x}-{{k}_{{\rm{net}},{\rm{max}}}}^{c = x}}{{{k}_{{\rm{net}},{\rm{max}}}}^{c = 6}-{{k}_{{\rm{net}},{\rm{max}}}}^{c = 1}}.$$

Therefore, the difference of the maximal net growth rate in the presence *k*_net,max,Δ_ and absence *k*_net,max_ at concentration *c* = *x* was normalized to the maximal possible change in the absence of interactions.

Further, a log fold change of the relative interaction effect was determined as:

14 $${\log }_{2}\left(\frac{{\rho }_{1}}{{\rho }_{2}}\right)$$

and used to compare the magnitude of the interaction effect in different scenarios.

**Table 2:** Model parameters and simulation settings

Description	Parameter	Value/Formula	Notes/References
Simulation settings			
Simulated time		4 h	A simulation time of 4 h is sufficient for populations to reach their maximal net growth rate.
Length of one timestep	Δ*t*	10 min	Simulations with shorter time steps provide approximately the same results when the system is simulated without interactions.
Number of iterations	–	50	50 iterations were found to be sufficient. The coefficient of variance between iterations showed no large changes when increasing the number of iterations further.
Initial species ratio	–	1:1	–
ODE parameters			
Bacteria			
Initial maximal replication rate	*k*_rep,max,0_	0.015 min^−1^	
Natural death rate	*k*_*d*0_	$\frac{0.2}{60}\,{\rm{min}}^{-1}$	^42^
Initial MIC	MIC_0_	6 mg ml^−1^	
Carrying capacity	*K*	$\frac{{N}_{{\rm{max}}}}{(1-\frac{{k}_{d0}}{{k}_{{\rm{rep}},{\rm{max}}}})}$	^40^
Maximal number of bacteria	*N*_max_	1000 bacteria	This value is based on a maximal population density of $1{0}^{9}\frac{{\rm{bacteria}}}{{\rm{ml}}}$ and the volume of the simulated space, 1 nanolitre.
Initial number of bacteria	*N*_init_	$\frac{1}{2}{N}_{{\rm{max}}}$ bacteria	
ABM parameters			
Interactions			
Interaction radius of short-range interactions	*r*	0	This setting represents interactions occurring at a distance of up to 100 μm between interacting bacteria^19^.
Interaction radius of long-range interactions	*r*	1	This setting represents interactions occurring at a distance of up to 200 μm between interacting bacteria.
Strength of negative MIC interaction at a long range	*f*_MIC,weak−_	−0.01	
	*f*_MIC,medium−_	−0.025	
	*f*_MIC,strong−_	−0.05	
Strength of positive MIC interaction at a long range	*f*_MIC,weak+_	0.01	
	*f*_MIC,medium+_	0.025	
	*f*_MIC,strong+_	0.05	
Strength of negative growth interaction (G) at a long range	*f*_*G*,weak−_	−0.039216	The strengths of growth interactions was determined to be equivalent to the strengths of MIC interactions.
	*f*_*G*,medium−_	−0.096923	
	*f*_*G*,strong−_	−0.189854	
Strength of positive growth interaction at a long range	*f*_*G*,weak+_	0.039771	
	*f*_*G*,medium+_	0.100387	
	*f*_*G*,strong+_	0.203657	
Strength of short-range interactions		*f*_*r* = 0_ = 9 * *f*_*r* = 1_	The strengths of short-range interactions are determined from the strengths of long-range interactions and corrected for the number of available patches in the interaction radius.
Range of growth interaction strengths	–	0; 3.5	The maximal strength causes a 3.5 fold change of the replication rate which is equivalent to a maximal replication rate of ${k}_{{\rm{rep}},{\rm{max}}}=0.0675\frac{1}{{\rm{min}}}$.
Range of interaction strengths for MIC− and MIC+	–	0; 3.5	The maximal strength causes a 3.5 fold change of the MIC which is equivalent to a maximal MIC of 27.
Grid and movement			
Dimensions of grid		12*12 patches	
Width and length of each patch		100 μm	
Height of grid		0.68 μm	This value is based on a microfluidic device^19^.
Number of steps per timestep for unstructured conditions	*n*_steps_	1	This setting represents an unstructured planktonic bacterial population of freely floating bacteria. The bacterial movement causes a high degree of mixing in the population. The number of steps per timestep approximately represents diffusion of bacteria with a diffusion coefficient of $2.6e-6\frac{{\rm{cm}}^{2}}{{\rm{s}}}$^47^.
Number of steps per timestep for structured conditions	*n*_steps_	0	This setting represents a structured biofilm population with restricted bacterial motility.

Parameters of the ordinary differential equation (ODE) model, as defined in Eqs. (3)–(9b)), describe characteristics and growth of bacterial populations. Parameters of the agent-based model (ABM), as defined in Eq. (2), describe interaction characteristics as well as the spatial grid and bacterial movement. All simulations were run with the defined simulation settings and the given parameter values. For two of the parameters, the number of steps and the interaction radius, multiple values are chosen representing different simulation scenarios.

### Reporting summary

Further information on research design is available in the Nature Research Reporting Summary linked to this article.

## Supplementary information

Reporting summary

## Data Availability

Simulation data were generated using custom scripts. A processed version of the data is available on GitHub in the vanhasseltlab/PolyMicro-ABM repository and on Zenodo at 10.5281/zenodo.10039816^45^ without restrictions.

## Code availability

The model was implemented in Python using the Mesa package^46^. All code necessary to reproduce the results is available on GitHub in the vanhasseltlab/PolyMicro-ABM repository and on Zenodo at 10.5281/zenodo.10039816^45^ without restrictions.

## References

[1] Peters, B. M., Jabra-Rizk, M. A., O’May, G. A., Costerton, J. W. & Shirtliff, M. E. Polymicrobial interactions: impact on pathogenesis and human disease. *Clin. Microbiol. Rev.* **25**, 193–213 (2012). 22232376 10.1128/CMR.00013-11 PMC3255964

[2] Radlinski, L. & Conlon, B. P. Antibiotic efficacy in the complex infection environment. *Curr. Opin. Microbiol.* **42**, 19–24 (2018). 28988156 10.1016/j.mib.2017.09.007 PMC5889345

[3] Brogden, K. A., Guthmiller, J. M. & Taylor, C. E. Human polymicrobial infections. *Lancet* **365**, 253–255 (2005). 15652608 10.1016/S0140-6736(05)17745-9 PMC7119324

[4] Anju, V. T. et al. Polymicrobial infections and biofilms: clinical significance and eradication strategies. *Antibiotics* **11**, 1731 (2022). 36551388 10.3390/antibiotics11121731 PMC9774821

[5] Bottery, M. J. et al. Inter-species interactions alter antibiotic efficacy in bacterial communities. *ISME J* **16**, 812–821 (2022). 34628478 10.1038/s41396-021-01130-6 PMC8857223

[6] De Vos, M. G., Zagorski, M., McNally, A. & Bollenbach, T. Interaction networks, ecological stability, and collective antibiotic tolerance in polymicrobial infections. *Proc. Natl Acad. Sci. USA* **114**, 10666–10671 (2017). 28923953 10.1073/pnas.1713372114 PMC5635929

[7] Mitra, S., Mallick, A. & Priyadarshini, S. Effect of polymicrobial interactions on antimicrobial resistance: an in vitro analysis in human ocular infections. *Future Microbiol.* **17**, 491–504 (2022). 35315292 10.2217/fmb-2021-0114

[8] Galera-Laporta, L. & Garcia-Ojalvo, J. Antithetic population response to antibiotics in a polybacterial community. *Sci. Adv.* **6**, eaaz5108 (2020). 32181369 10.1126/sciadv.aaz5108 PMC7060062

[9] Bottery, M. J., Pitchford, J. W. & Friman, V.-P. Ecology and evolution of antimicrobial resistance in bacterial communities. *ISME J.* **15**, 939–948 (2021). 33219299 10.1038/s41396-020-00832-7 PMC8115348

[10] Estrela, S. & Brown, S. P.Community interactions and spatial structure shape selection on antibiotic resistant lineages. *PLoS Comput. Biol.* **14**, e1006179 (2018). 29927925 10.1371/journal.pcbi.1006179 PMC6013025

[11] Adamowicz, E. M., Muza, M., Chacón, J. M. & Harcombe, W. R. Cross-feeding modulates the rate and mechanism of antibiotic resistance evolution in a model microbial community of Escherichia coli and Salmonella enterica. *PLoS Pathog* **16**, e1008700 (2020). 32687537 10.1371/journal.ppat.1008700 PMC7392344

[12] Mizrahi, S. P., Goyal, A. & Gore, J. Community interactions drive the evolution of antibiotic tolerance in bacteria. *Proc. Natl. Acad. Sci. USA* **120**, e2209043119 (2023). 36634144 10.1073/pnas.2209043119 PMC9934204

[13] Armbruster, C. E. et al. Indirect pathogenicity of haemophilus influenzae and Moraxella catarrhalis in polymicrobial otitis media occurs via interspecies quorum signaling. *mBio* **1**, e00102–10 (2010). 20802829 10.1128/mBio.00102-10 PMC2925075

[14] Filkins, L. M. & O’Toole, G. A. Cystic fibrosis lung infections: polymicrobial, complex, and hard to treat. *PLoS Pathog.* **11**, e1005258 (2015). 26719892 10.1371/journal.ppat.1005258 PMC4700991

[15] Rolston, K. V. I., Bodey, G. P. & Safdar, A. Polymicrobial infection in patients with cancer: an underappreciated and underreported entity. *Clin. Infect. Dis.* **45**, 228–233 (2007). 17578784 10.1086/518873

[16] Klotz, S. A., Chasin, B. S., Powell, B., Gaur, N. K. & Lipke, P. N. Polymicrobial bloodstream infections involving Candida species: analysis of patients and review of the literature. *Diagn. Microbiol. Infect. Dis.* **59**, 401–406 (2007). 17888612 10.1016/j.diagmicrobio.2007.07.001

[17] Dowd, S. E. et al. Survey of bacterial diversity in chronic wounds using Pyrosequencing, DGGE, and full ribosome shotgun sequencing. *BMC Microbiol.* **8**, 43 (2008). 18325110 10.1186/1471-2180-8-43 PMC2289825

[18] Ronald, A. The etiology of urinary tract infection: traditional and emerging pathogens. *Am. J. Med.* **113**, 14–19 (2002). 10.1016/s0002-9343(02)01055-0 12113867

[19] Dal Co, A., van Vliet, S., Kiviet, D. J., Schlegel, S. & Ackermann, M. Short-range interactions govern the dynamics and functions of microbial communities. *Nat. Ecol. Evol.* **4**, 366–375 (2020). 32042125 10.1038/s41559-019-1080-2

[20] van Vliet, S., Hauert, C., Fridberg, K., Ackermann, M. & Co, A. D. Global dynamics of microbial communities emerge from local interaction rules. *PLoS Comput. Biol.* **18**, e1009877 (2022). 35245282 10.1371/journal.pcbi.1009877 PMC8926250

[21] Flynn, J. M. et al. Disruption of cross-feeding inhibits pathogen growth in the sputa of patients with cystic fibrosis. *mSphere* **5**, e00343–20 (2020). 32350096 10.1128/mSphere.00343-20 PMC7193046

[22] Perlin, M. H. et al. Protection of Salmonella by ampicillin-resistant Escherichia coli in the presence of otherwise lethal drug concentrations. *Proc. R. Soc. B Biol. Sci.* **276**, 3759–3768 (2009). 10.1098/rspb.2009.0997 PMC2817278 19656787

[23] Sorg, R. A. et al. Collective resistance in microbial communities by intracellular antibiotic deactivation. *PLoS Biol.* **14**, e2000631 (2016). 28027306 10.1371/journal.pbio.2000631 PMC5189934

[24] Beaudoin, T. et al. Staphylococcus aureus interaction with Pseudomonas aeruginosa biofilm enhances tobramycin resistance. *npj Biofilms Microbiomes* **3**, 1–9 (2017). 29062489 10.1038/s41522-017-0035-0 PMC5648753

[25] Aranda-Díaz, A. et al. Bacterial interspecies interactions modulate pH-mediated antibiotic tolerance. *eLife* **9**, e51493 (2020). 31995029 10.7554/eLife.51493 PMC7025823

[26] DeLeon, S. et al. Synergistic interactions of Pseudomonas aeruginosa and Staphylococcus aureus in an in vitro wound model. *Infect. Immun.* **82**, 4718–4728 (2014). 25156721 10.1128/IAI.02198-14 PMC4249327

[27] Armbruster, C. R. et al. Staphylococcus aureus protein A mediates interspecies interactions at the cell surface of Pseudomonas aeruginosa. *mBio* **7**, e00538-16 (2016). 27222468 10.1128/mBio.00538-16 PMC4895107

[28] Granato, E. T., Meiller-Legrand, T. A. & Foster, K. R. The evolution and ecology of bacterial warfare. *Curr. Biol.* **29**, R521–R537 (2019). 31163166 10.1016/j.cub.2019.04.024

[29] van den Berg, N.I., Machado, D. & Santos, S. et al. Ecological modelling approaches for predicting emergent properties in microbial communities. *Nat Ecol Evol.* **6**, 855–865 (2022). 35577982 10.1038/s41559-022-01746-7 PMC7613029

[30] Nguyen, A. T. & Oglesby-Sherrouse, A. G. Interactions between Pseudomonas aeruginosa and Staphylococcus aureus during co-cultivations and polymicrobial infections. *Appl. Microbiol. Biotechnol.* **100**, 6141–6148 (2016). 27236810 10.1007/s00253-016-7596-3 PMC4916000

[31] Stacy, A., McNally, L., Darch, S. E., Brown, S. P. & Whiteley, M. The biogeography of polymicrobial infection. *Nat. Rev. Microbiol.* **14**, 93–105 (2016). 26714431 10.1038/nrmicro.2015.8 PMC5116812

[32] Marcus, G. et al. Intra-abdominal infections: the role of anaerobes, enterococci, fungi, and multidrug-resistant organisms. *Open Forum Infect. Dis.* **3**, ofw232 (2016). 28018930 10.1093/ofid/ofw232 PMC5170494

[33] Booth, S. C. & Rice, S. A. Influence of interspecies interactions on the spatial organization of dual species bacterial communities. *Biofilm* **2**, 100035 (2020). 33447820 10.1016/j.bioflm.2020.100035 PMC7798468

[34] Cordero, O. X. & Datta, M. S. Microbial interactions and community assembly at microscales. *Curr. Opin. Microbiol.* **31**, 227–234 (2016). 27232202 10.1016/j.mib.2016.03.015 PMC5157693

[35] Barraza, I. et al. Disturbing the spatial organization of biofilm communities affects expression of agr-regulated virulence factors in Staphylococcus aureus. *Appl. Environ. Microbiol.* **0**, e01932–22 (2023). 10.1128/aem.01932-22 PMC9973005 36700647

[36] Wang, J. et al. Psl-dependent cooperation contributes to drug resistance of Pseudomonas aeruginosa in dual-species biofilms with Acinetobacter baumannii. *ACS Infect. Dis.* **8**, 129–136 (2022). 34936325 10.1021/acsinfecdis.1c00416

[37] Wilson, C. E. et al. Cooperation and competition shape ecological resistance during periodic spatial disturbance of engineered bacteria. *Sci. Rep.* **7**, 440 (2017). 28348396 10.1038/s41598-017-00588-9 PMC5428654

[38] Nel Van Zyl, K., Matukane, S. R., Hamman, B. L., Whitelaw, A. C. & Newton-Foot, M. Effect of antibiotics on the human microbiome: a systematic review. *Int. J. Antimicrob. Agents* **59**, 106502 (2022). 34929293 10.1016/j.ijantimicag.2021.106502

[39] Patangia, D. V., Anthony Ryan, C., Dempsey, E., Paul Ross, R. & Stanton, C. Impact of antibiotics on the human microbiome and consequences for host health. *MicrobiologyOpen* **11**, e1260 (2022). 35212478 10.1002/mbo3.1260 PMC8756738

[40] Stressmann, F. A. et al. Does bacterial density in cystic fibrosis sputum increase prior to pulmonary exacerbation? *J. Cyst. Fibros.* **10**, 357–365 (2011). 21664196 10.1016/j.jcf.2011.05.002

[41] Czock, D. & Keller, F. Mechanism-based pharmacokinetic-pharmacodynamic modeling of antimicrobial drug effects. *J. Pharmacokinet. Pharmacodyn.* **34**, 727–751 (2007). 17906920 10.1007/s10928-007-9069-x

[42] Nielsen, E. I. & Friberg, L. E. Pharmacokinetic-pharmacodynamic modeling of antibacterial drugs. *Pharmacol. Rev.* **65**, 1053–1090 (2013). 23803529 10.1124/pr.111.005769

[43] Regoes, R. R. et al. Pharmacodynamic functions: a multiparameter approach to the design of antibiotic treatment regimens. *Antimicrob. Agents Chemother.* **48**, 3670–3676 (2004). 15388418 10.1128/AAC.48.10.3670-3676.2004 PMC521919

[44] Baranyi, J. & Roberts, T. A. Mathematics of predictive food microbiology. *Int. J. Food Microbiol.* **26**, 199–218 (1995). 7577358 10.1016/0168-1605(94)00121-l

[45] Herzberg, C. Vanhasseltlab/PolyMicro-ABM: PolyMicro-ABM. Zenodo (2023).

[46] Kazil, J., Masad, D. & Crooks, A. Utilizing python for agent-based modeling: the mesa framework. In *Social, Cultural, and Behavioral Modeling* (eds Thomson, R., Bisgin, H., Dancy, C., Hyder, A. & Hussain, M.) 308–317 (Springer International Publishing, 2020).

[47] Berg, H. C. & Turner, L. Chemotaxis of bacteria in glass capillary arrays. Escherichia coli, motility, microchannel plate, and light scattering. *Biophys. J.* **58**, 919–930 (1990). 2248995 10.1016/S0006-3495(90)82436-X PMC1281037

